# Ocular Pigmentation Impact on Retinal Versus Choroidal Optical Coherence Tomography Imaging in Preterm Infants

**DOI:** 10.1167/tvst.12.7.7

**Published:** 2023-07-06

**Authors:** Kai R. Seely, Michelle McCall, Gui-Shuang Ying, S. Grace Prakalapakorn, Sharon F. Freedman, Cynthia A. Toth, for the BabySTEPS Group

**Affiliations:** 1 Department of Ophthalmology, Duke University, Durham, NC, USA; 2 Center for Preventive Ophthalmology and Biostatistics, University of Pennsylvania, Philadelphia, PA, USA; 3 Department of Pediatrics, Duke University, Durham, NC, USA; 4 Department of Biomedical Engineering, Duke University, Durham, NC, USA

**Keywords:** optical coherence tomography, retinopathy of prematurity, retinal imaging

## Abstract

**Purpose:**

To evaluate the association of fundus pigmentation with the visibility of retinal versus choroidal layers on optical coherence tomography (OCT) in preterm infants.

**Methods:**

For infants enrolled in BabySTEPS, ophthalmologists recorded fundus pigmentation (blond, medium, or dark) at the first retinopathy of prematurity (ROP) examination. Bedside OCT imaging was performed at each examination, and a masked grader evaluated all OCT scans from both eyes of each infant for visibility (yes/no) of all retinal layers and of the chorio–scleral junction (CSJ). Multivariable logistic regression was used to assess associations between fundus pigmentation and visibility of all retinal layers and CSJ, controlling for potential confounders (i.e., birth weight, gestational age, sex, OCT system, pupil size, and postmenstrual age at imaging).

**Results:**

Among 114 infants (mean birth weight, 943 grams; mean gestational age, 27.6 weeks), 43 infants (38%) had blond, 56 infants (49%) had medium, and 15 infants (13%) had dark fundus pigmentation. Of 1042 scans, all retinal layers were visible in 977 (94%) and CSJ in 895 (86%). Pigmentation was not associated with retinal layer visibility (*P* = 0.49), but medium and dark pigmentation were associated with decreased CSJ visibility (medium: odds ratio [OR] = 0.34, *P* = 0.001; dark: OR = 0.24, *P* = 0.009). For infants with dark pigmentation, retinal layer visibility increased (OR = 1.87 per week; *P* ≤ 0.001) and CSJ visibility decreased (OR = 0.78 per week; *P* = 0.01) with increasing age.

**Conclusions:**

Although fundus pigmentation was not associated with the visibility of all retinal layers on OCT, darker pigmentation decreased CSJ visibility, and this effect increased with age.

**Translational Relevance:**

The ability of bedside OCT to capture retinal layer microanatomy in preterm infants, regardless of fundus pigmentation, may represent an advantage over fundus photography for ROP telemedicine.

## Introduction

Retinopathy of prematurity (ROP) is a leading cause of childhood blindness worldwide. The gold standard for ROP screening is an examination by a trained ophthalmologist using indirect ophthalmoscopy, with fundus photography emerging as a potential adjunct.^1^ However, widefield fundus photography has several limitations, including that bright visible light may be stressful to preterm infants,^2^^,^^3^ hazy media can obscure images in infants <33 weeks’ postmenstrual age (PMA),^4^^,^^5^ poor pupil dilation can darken the image, and inadvertent corneal pressure can change the appearance of ROP severity.^6^^,^^7^ Additionally, visualization of ROP on fundus photography can be impacted by fundus pigmentation^8^^,^^9^—specifically, it may be confounded by choroidal vasculature in eyes with blond pigmentation^10^ and limited by low contrast in eyes with dark pigmentation.^4^^,^^11^

Optical coherence tomography (OCT) may be a future, potential, alternative imaging modality for ROP. Research using investigational non-contact bedside OCT has identified subclinical microanatomic changes in the retinas of preterm infants, including macular edema,^12^ photoreceptor immaturity,^13^ and abnormal retinal and choroidal layer thicknesses.^14^^,^^15^ In preterm infants, OCT has also been used to visualize ROP stage at the vascular–avascular junction,^16^^–^^18^ as well as the dilated and tortuous posterior pole vessels of pre-plus and plus disease.^19^^,^^20^ Non-contact OCT imaging is also less stressful to preterm infants than contact fundus photography.^21^ However, it is not known whether bedside OCT imaging in preterm infants is impacted by fundus pigmentation.

Fundus pigmentation is the result of melanin deposition in two histologic layers, the retinal pigment epithelium (RPE) and the choroid.^22^^,^^23^ Pigmentation in the RPE develops early in gestation and does not vary by race, whereas choroidal pigmentation develops later (in the third trimester) and is more prominent in individuals with darker skin.^22^^,^^23^ Melanin in these two pigmented layers reflects or absorbs the near-infrared light used in OCT imaging, potentially limiting penetration of the signal to deeper structures.^24^^–^^26^ One previous study of 86 preterm infants assessed the impact of maternal identification of race on OCT imaging of the choroid but did not investigate fundus pigmentation as a relevant biological variable.^27^ They found that maternal self-identification as African American race was associated with decreased chorio–scleral junction (CSJ) visibility among term-age preterm infants.

The purpose of this study was to evaluate the association between fundus pigmentation and visibility of retinal versus choroidal layers on bedside OCT in preterm infants. We hypothesized that medium and dark fundus pigmentation would be associated with decreased CSJ visibility, but not with decreased retinal layer visibility.

## Methods

This is an analysis of OCT images captured between November 2016 and March 2021 under BabySTEPS (NCT02887157), a prospective observational study of retinal microanatomy in ROP. BabySTEPS was approved by the Duke University Health System Institutional Review Board and adhered to the tenets of the Declaration of Helsinki, Good Clinical Practice, and the Health Insurance Portability and Accountability Act, and each participant’s parent or guardian provided written informed consent.

As part of BabySTEPS, we imaged both eyes of each infant on the same day as standard-of-care clinical ROP examinations using investigational, bedside, swept-source OCT. Two ultra-compact handheld OCT probes were used for imaging: (1) 100-kHz OCT system centered at 1047 nm, with a handpiece that captured 512 A-scans per B-scan and 112 B-scans per 6 × 6-mm volume (UC2; November 2016–December 2018)^28^; and (2) a 1060-nm, 200-kHz probe that captured 950 A-scans per B-scan and 256 B-scans per 10 × 10-mm volume (UC3; December 2018–March 2021).^29^ OCT imagers measured pupil size prior to imaging in ambient light using black dots for scale and prioritized capture of the foveal center.

**Figure 1.**
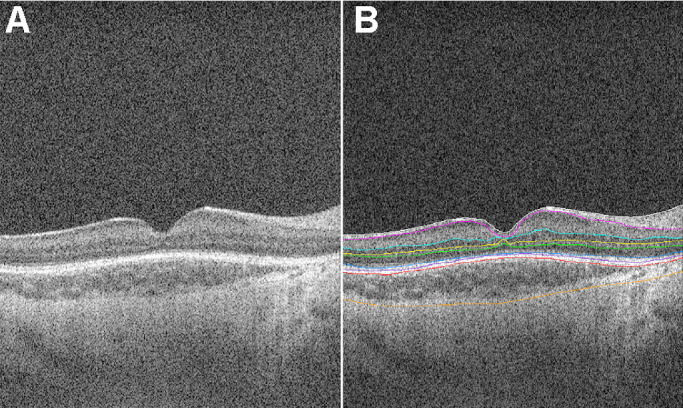
Foveal OCT scan captured from a preterm infant at the bedside. (**A**) OCT scan without layer segmentation; (**B**) OCT scan with all age-appropriate retinal layers and the CSJ semiautomatically segmented. Color codes, from inner to outer: *white*, internal limiting membrane; *magenta*, retina nerve fiber layer/ganglion cell layer; *cyan*, inner plexiform layer/inner nuclear layer; *yellow*, inner nuclear layer/outer plexiform layer; *green*, outer plexiform layer/outer nuclear layer; *blue*, inner segment/outer segment; *purple*, outer segment/retinal pigment epithelium; *red*, Bruch's membrane; *orange*, CSJ.

Custom infant-specific software, the Duke OCT Retinal Analysis Program Marking Code (DOCTRAP, MATLAB R2017b; MathWorks, Natick, MA), was used to autosegment retinal layers on foveal OCT volumes (Fig. 1). As part of the primary analysis for BabySTEPS, an experienced OCT grader assessed the following: (1) visibility of all age-appropriate retinal layers (including retinal nerve fiber layer, ganglion cell layer, inner plexiform layer, inner nuclear layer, outer plexiform layer, outer nuclear layer, and retinal pigment epithelium); and (2) visibility of the CSJ. We decided not to analyze the visibility of the ellipsoid zone as an OCT outcome in this study, as the ellipsoid zone is absent in a significant number of preterm infants.^30^ OCT imagers and graders were masked to demographic and clinical features, including fundus pigmentation. During standard-of-care ophthalmoscopic examinations, a fellowship-trained pediatric ophthalmologist graded fundus pigmentation as blond, medium, or dark using previously published fundus photographs as reference.^31^ For infants who had two different fundus pigmentations recorded during the nursery stay (*n* = 4), the most frequently recorded fundus pigmentation was used for analysis. Clinicians were masked to OCT findings.

We included all infants that had fundus pigmentation recorded as part of BabySTEPS and did not have ocular pathology that precluded OCT imaging (e.g., persistent tunica vasculosa lentis, vitreous hemorrhage, poor corneal clarity, cataract). For each included infant, we analyzed all OCT imaging sessions of both eyes that occurred between 30 and 42 weeks’ PMA, that captured the foveal center, and that occurred prior to any ROP treatment. We extracted the following variables from the clinical chart: birth weight, gestational age, sex, and PMA at OCT imaging. The maternal identification of race (American Indian or Alaska Native, Asian, Black or African American, Native Hawaiian or Other Pacific Islander, White, or Multiple) and maternal ethnicity (Hispanic or non-Hispanic) were collected for this National Institute of Health–funded project following the racial and ethnic standards set by the Office of Management and Budget.^32^^,^^33^ We classified infants by the maternal identification of race and binarized maternal race as “White” versus “non-White” for analysis because only four infants were categorized as Asian, four as Multiple, and none as American Indian or Alaska Native or Native Hawaiian or Other Pacific Islander.

We performed all statistical analyses using R 3.6.1 (R Foundation for Statistical Computing, Vienna, Austria). We performed Fisher’s exact test for comparison of categorical variables between the fundus pigmentation groups and performed ANOVA for comparison of continuous variables among the fundus pigmentation groups. The proportion of OCT scans with all retinal layers at each 2-week PMA window (i.e., 30–32 weeks, 32–34 weeks, 34–36 weeks, 36–38 weeks, 38–40 weeks, and 40–42 weeks) was calculated, both overall and for each pigmentation group. These proportions were also calculated with regard to CSJ visibility. We performed multivariable logistic regression to evaluate the association between fundus pigmentation (blonde, medium, or dark) and the following OCT outcomes: (1) all age-appropriate retinal layers visible (yes vs. no), and (2) CSJ visible (yes vs. no). We adjusted for birth weight, gestational age, PMA at OCT imaging, pupil size at OCT imaging, and OCT system (UC2 or UC3) used. We used generalized estimating equations to account for repeated-measures correlation and inter-eye correlation. Each feature was first evaluated in a univariable logistic regression model; those that were statistically significantly associated with OCT parameters were then included in a multivariable logistic regression model. Fundus pigmentation, the primary variable of interest, was included into each multivariable regression model, no matter whether it was associated with OCT parameters or not in the univariable logistic regression model. We performed a test of interaction between fundus pigmentation and age at OCT imaging, and when there was significant interaction we performed analysis of association between age at OCT imaging stratified by fundus pigmentation with regard to OCT outcomes. For these analyses, we again adjusted for clinical factors that were statistically significant on univariable regression analysis. A two-sided *P* ≤ 0.05 was considered as statistical significance for all analyses.

## Results

We included 114 of 122 infants (93%) enrolled in BabySTEPS. The mean ± SD gestational age was 27.6 ± 2.6 weeks, mean birth weight was 943 ± 280 grams, and mean imaging sessions per infant were 4.7 ± 2.6 (Table 1). From the 114 included infants, we analyzed investigational foveal OCT scans from 1042 imaging sessions. We excluded four infants because they did not have fundus pigmentation recorded during the nursery stay and four because they did not undergo investigational OCT imaging between 30 and 42 weeks’ PMA. Of the four infants without OCT data, one had blond fundus pigmentation and three had medium fundus pigmentation.

**Table 1.** Demographic and Clinical Characteristics of Preterm Infants Overall and by Fundus Pigmentation

	Overall *N* = 114 (100%)	Blond *N* = 43 (38%)	Medium *N* = 56 (49%)	Dark *N* = 15 (13%)	*P* ^a^
Gestational age (wk), mean ± SD	27.6 ± 2.6	27.3 ± 2.6	27.6 ± 2.7	27.8 ± 2.2	0.51
Birth weight (g), mean ± SD	943 ± 280	913 ± 319	947 ± 252	1012 ± 269	0.49
Male sex, *n* (%)	49 (52)	25 (58)	29 (52)	5 (33)	0.26
Pupil size at OCT imaging (mm), mean ± SD	5.2 ± 1.2	5.3 ± 1.2	5.3 ± 1.3	5.0 ± 1.3	0.022
Maternal race, *n* (%)					
White	52 (46)	35 (81)	17 (30)	0 (0)	<0.001
Non-White	62 (54)	8 (19)	39 (70)	15 (100)	<0.001
African American	54 (47)	6 (14)	34 (61)	14 (93)	
Asian	4 (4)	1 (3)	3 (5)	0 (0)	
Multiple	4 (4)	1 (3)	2 (4)	1 (7)	
Maternal Hispanic ethnicity, *n* (%)	10 (9)	4 (9)	5 (9)	1 (7)	1.000
Number of imaging sessions per infant, mean ± SD	4.7 ± 2.6	5.1 ± 2.7	4.5 ± 2.6	4.2 ± 2.4	0.34
PMA at first OCT imaging session (wk), mean ± SD	33.1 ± 2.0	32.8 ± 1.8	33.2 ± 2.2	33.5 ± 1.3	0.47
PMA at last OCT imaging session (wk), mean ± SD	38.9 ± 2.6	39.2 ± 2.5	38.6 ± 2.4	38.8 ± 3.3	0.43

a *P* values were obtained from Fisher's exact test for comparison of categorical variables and ANOVA for comparison of means of continuous variables.

Overall, 43 infants (38%) had blond, 56 infants (49%) had medium, and 15 infants (13%) had dark fundus pigmentation (Table 1). Fundus pigmentation was consistent across all examinations for 110 infants (96%), and fundus pigmentation had 100% inter-eye agreement. Among the four infants whose pigment varied between examinations, three were found to have medium pigmentation at one examination and dark pigmentation at the other examinations (and were therefore considered to have dark pigmentation for analysis); the fourth infant was found to have blond pigmentation at one examination and medium pigmentation at the other examinations (and was therefore considered to have medium pigmentation for analysis). Mean gestational age, birth weight, and number of imaging sessions per infant were similar among infants with blond, medium, and dark fundus pigmentation (Table 1). Pupil size at time of imaging ranged from 1.0 mm to 8.0 mm, and mean pupil size was smaller in infants with dark fundus pigmentation (5.0 mm) than in infants with blond or medium pigmentation (5.3 mm and 5.3 mm, respectively; *P* = 0.022) (Table 1). Fundus pigmentation was associated with maternal identification of race: Infants with blond pigmentation were much more likely to be White (81%) than were those with medium or dark pigmentation (30% and 0%, respectively; *P* < 0.001) (Table 1).

All retinal layers were visible in 977 OCT scans (94%) (Table 2). On univariable logistic regression analysis, male sex (odds ratio [OR] = 2.58; *P* = 0.029), use of the UC3 probe (OR = 3.06; *P* = 0.048), and greater birth weight (OR = 1.24 per 100-gram increase; *P* = 0.003) were associated with increased odds of all retinal layers being visible. When these factors were included in multivariable logistic regression analysis, male sex (OR = 2.93; *P* = 0.011), use of the UC3 probe (OR = 4.42; *P* = 0.007), and greater birth weight (OR = 1.28 per 100-gram increase; *P* < 0.001) all remained statistically significantly associated with visibility of all retinal layers. Fundus pigmentation was not associated with visibility of all retinal layers on univariable (*P* = 0.49) or multivariable (*P* = 0.49) logistic regression analysis.

**Table 2.** Univariable and Multivariable Logistic Regression Analysis for Factors Associated With Visibility of All Retinal Layers on OCT

	All Retinal Layers Visible (977/1042; 94%)	Unadjusted OR (95% CI)	*P*	Adjusted OR (95% CI)^a^	*P*
Fundus pigmentation			0.49		0.49
Blond	398 (93%)	Ref.	—	Ref.	—
Medium	461 (94%)	1.12 (0.38–3.28)	0.84	0.83 (0.31–2.26)	0.72
Dark	118 (97%)	2.22 (0.55–9.08)	0.27	2.13 (0.55–8.18)	0.27
Male sex	—	2.58 (1.10–6.03)	0.029	2.93 (1.28–6.73)	0.011
UC3 probe^b^	—	3.06 (1.01–9.26)	0.048	4.42 (1.50–12.98)	0.007
Gestational age, per 1-week increase	—	1.05 (0.86–1.28)	0.63	—	—
Birth weight, per 100-g increase	—	1.24 (1.08–1.44)	0.003	1.28 (1.11–1.47)	<0.001
Pupil size at OCT imaging, per 1-mm increase	—	1.14 (0.87–1.48)	0.34	—	—
PMA at OCT imaging, per 1-week increase	—	1.11 (0.95–1.30)	0.17	—	—

a Multivariable analysis was adjusted by fundus pigmentation and factors significant in univariable analysis.

b The UC3 probe was a 1060-nm, 200-kHz ultra-compact handheld OCT probe that captured 950 A-scans per B-scan and 256 B-scans per 10 × 10-mm volume.

The CSJ was visible in 895 OCT scans (86%) (Table 3). On univariable logistic regression analysis, use of the UC3 probe (OR = 17.7; *P* < 0.001) was associated with increased odds of the CSJ being visible, whereas greater gestational age (OR = 0.87 per 1-week increase; *P* = 0.04), greater birth weight (OR = 0.88 per 100-gram increase; *P* = 0.03), and medium (OR = 0.47; *P* = 0.03) and dark (OR = 0.24; *P* = 0.03) fundus pigmentation were associated with decreased odds of CSJ being visible. When these factors were included in a multivariable logistic regression model, use of the UC3 probe (OR = 20.8; *P* < 0.001) and medium (OR = 0.34; *P* = 0.001) and dark (OR = 0.24; *P* = 0.009) fundus pigmentation remained statistically significantly associated with CSJ visibility.

**Table 3.** Univariable and Multivariable Logistic Regression Analysis for Factors Associated With CSJ Visibility on OCT

Factor	CSJ Visible (895/1042; 86%)	Unadjusted OR (95% CI)	*P*	Adjusted OR (95% CI)^a^	*P*
Fundus pigmentation			0.02		0.001
Blond	392 (92%)	Ref.	—	Ref.	—
Medium	412 (84%)	0.47 (0.24–0.92)	0.03	0.34 (0.17–0.65)	0.001
Dark	91 (75%)	0.24 (0.09–0.84)	0.03	0.24 (0.08–0.70)	0.009
Male sex	—	1.59 (0.80–3.15)	0.19	—	—
UC3 probe^b^	—	17.73 (7.73–40.66)	<0.001	20.76 (9.38–45.90)	<0.001
Gestational age, per 1-week increase	—	0.87 (0.77–0.99)	0.04	0.87 (0.72–1.06)	0.16
Birth weight, per 100-g increase	—	0.88 (0.78–0.99)	0.03	1.00 (0.87–1.15)	0.99
Pupil size at OCT imaging, per 1-mm increase	—	1.11 (0.93–1.32)	0.24	—	—
PMA at OCT imaging, per 1-week increase	—	1.01 (0.94–1.10)	0.75	—	—

a Multivariable analysis was adjusted by fundus pigmentation and factors significant in univariable analysis.

b The UC3 probe was a 1060-nm, 200-kHz ultra-compact handheld OCT probe that captured 950 A-scans per B-scan and 256 B-scans per 10 × 10-mm volume.

**Figure 2.**
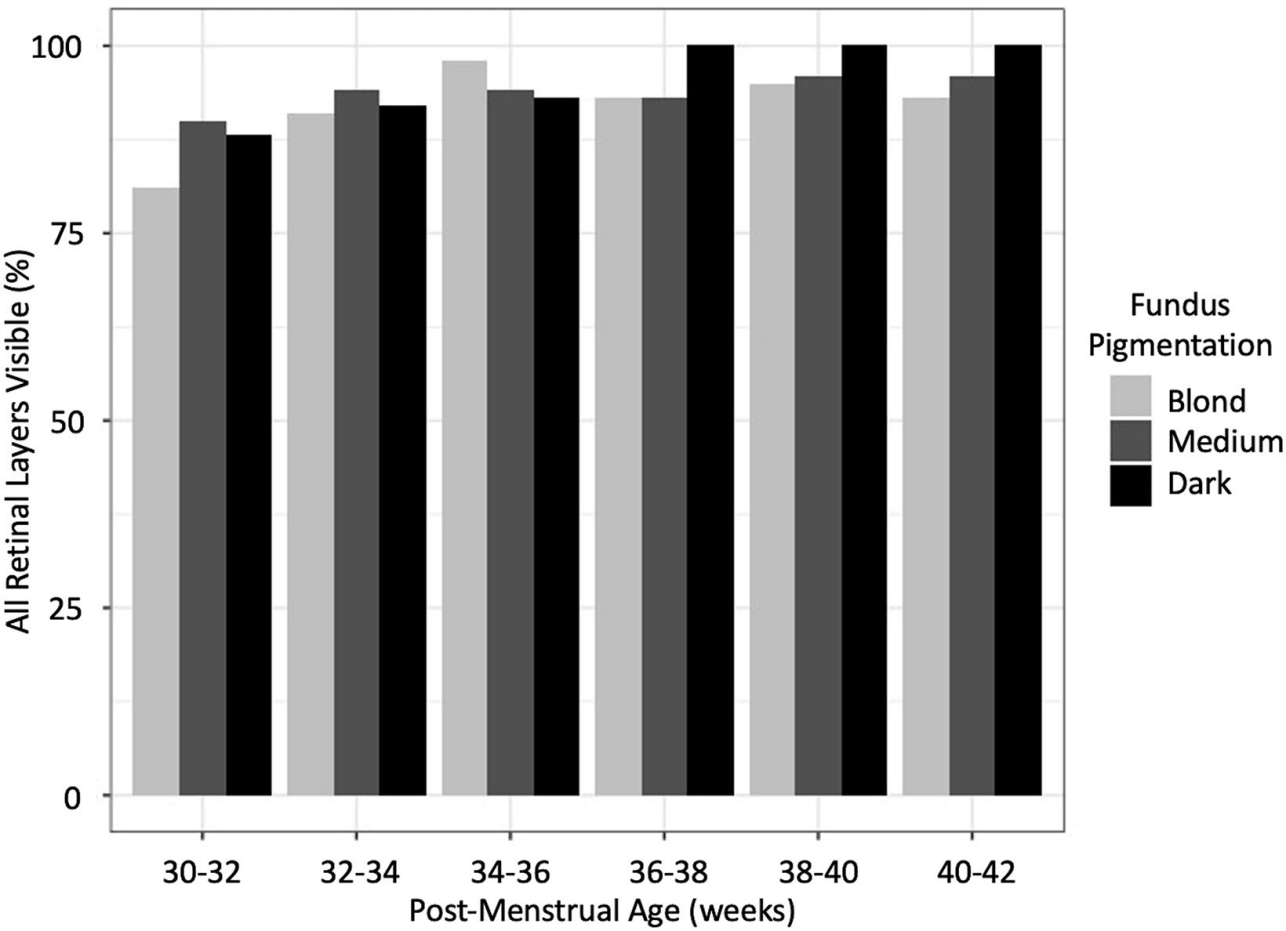
Proportion of foveal OCT scans, captured at the bedside in preterm infants, with all retinal layers visible, during each 2-week age window by fundus pigmentation. In infants with dark fundus pigmentation, the odds of all retinal layers being visible significantly increased with increasing age (adjusted OR = 1.87 per 1-week increase; *P* < 0.001).

**Figure 3.**
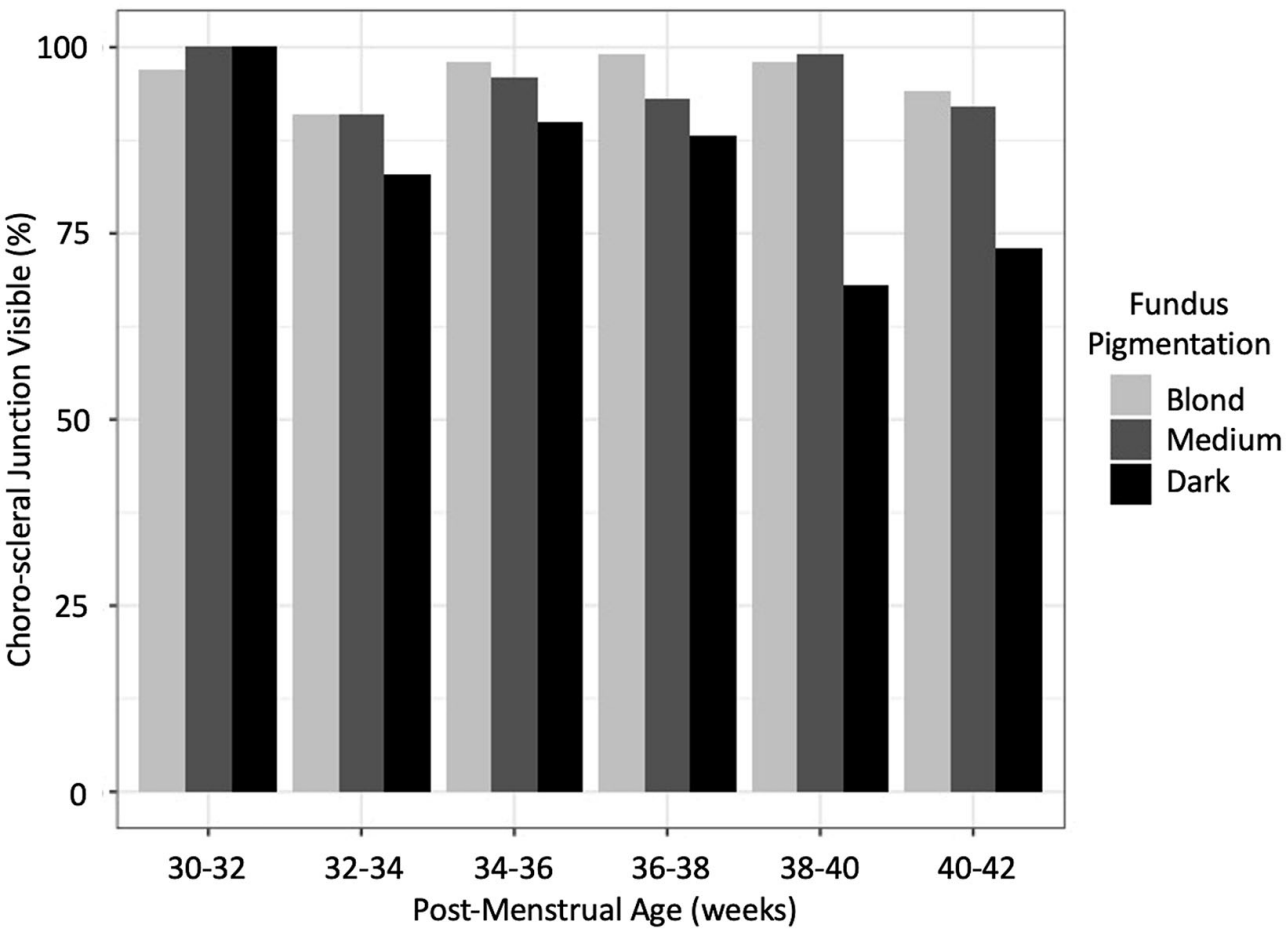
Proportion of foveal OCT scans, captured at the bedside in preterm infants, with the CSJ visible, during each 2-week age window by fundus pigmentation. In infants with dark fundus pigmentation, the odds of the CSJ being visible significantly decreased with increasing age (adjusted OR = 0.78 per 1-week increase; *P* = 0.01).

When subanalyzed by pigmentation and age at imaging, retinal layer visibility was >80% in all groups (Table 4, Fig. 2). The lowest visibility was among infants with blond pigmentation at less than 32 weeks’ PMA (81%), and the highest visibility was among infants with dark pigmentation between 36 and 38, 38 and 40, and 40 and 42 weeks (all 100%) (Table 4, Fig. 2). Notably, CSJ visibility was >90% at all ages among infants with blond or medium pigmentation, but was lower among those with dark pigmentation, especially at older ages (the lowest visibility being 68% among infants with dark pigmentation at 38–40 weeks’ PMA) (Table 5, Fig. 3). There was statistically significant interaction between fundus pigmentation and age at OCT imaging with regard to both visibility of all retinal layers and CSJ (*P* < 0.001 and *P* = 0.005, respectively), although in opposite directions. In the stratified analysis by fundus pigmentation, there was no association between age at OCT imaging and OCT outcomes among infants with blond or medium pigmentation. However, among infants with dark pigmentation, older age at imaging was statistically significantly associated with increased visibility of all retinal layers (adjusted OR = 1.87 per 1-week increase; *P* < 0.001) and decreased CSJ visibility (adjusted OR = 0.78 per 1-week increase; *P* = 0.01) (Table 6).

**Table 4.** Proportion of Foveal OCT Scans Captured At the Bedside in Preterm Infants With All Retinal Layers Visible During Each 2-Week Age Window by Fundus Pigmentation

	Pigmentation			
PMA (wk)	Blond	Medium	Dark	Total
30–32	29/36 (81%)	43/48 (90%)	7/8 (88%)	79/92 (86%)
32–34	58/64 (91%)	89/95 (94%)	11/12 (92%)	158/171 (92%)
34–36	92/94 (98%)	107/114 (94%)	28/30 (93%)	227/238 (95%)
36–38	82/88 (93%)	102/110 (93%)	25/25 (100%)	209/223 (94%)
38–40	71/75 (95%)	73/76 (96%)	25/25 (100%)	169/183 (92%)
40–42	66/71 (93%)	47/49 (96%)	22/22 (100%)	135/142 (95%)
Total	398/428 (93%)	461/492 (94%)	118/122 (97%)	977/1042 (94%)

In infants with dark fundus pigmentation, the odds of all retinal layers being visible significantly increased with increasing age (adjusted OR = 1.87 per 1-week increase; *P* < 0.001). However, visibility at the youngest ages (i.e., less than 32 weeks’ PMA) among infants with dark pigmentation was similar to or greater than that in infants with blond or medium pigmentation.

**Table 5.** Proportion of Foveal OCT Scans Captured at the Bedside in Preterm Infants With the CSJ Visible During Each 2-Week Age Window by Fundus Pigmentation

	Pigmentation			
PMA (wk)	Blond	Medium	Dark	Total
30–32	35/36 (97%)	48/48 (100%)	8/8 (100%)	91/92 (99%)
32–34	58/64 (91%)	86/95 (91%)	10/12 (83%)	154/171 (90%)
34–36	92/94 (98%)	109/114 (96%)	27/30 (90%)	228/238 (96%)
36–38	87/88 (99%)	102/110 (93%)	22/25 (88%)	211/223 (95%)
38–40	73/75 (98%)	75/76 (99%)	17/25 (68%)	165/183 (90%)
40–42	67/71 (94%)	45/49 (92%)	16/22 (73%)	128/142 (90%)
Total	412/428 (96%)	465/492 (95%)	100/122 (82%)	977/1042 (94%)

In infants with dark fundus pigmentation, the odds of the CSJ being visible significantly decreased with increasing age (adjusted OR = 0.78 per 1-week increase; *P* = 0.01). Visibility was lower among infants with dark pigmentation than in those with blond or medium pigmentation, especially at older ages (i.e., greater than 38 weeks’ PMA).

**Table 6.** Multivariable Logistic Regression Analysis Stratified by Fundus Pigmentation for Association of Age at OCT Imaging With Retinal Layer and CSJ Visibility on OCT

Factor, Age at OCT Imaging, Per 1-Week Increase	Adjusted OR of All Retinal Layers Visible (95% CI)^a^	*P*	Adjusted OR of CSJ Visible (95% CI)^b^	*P*
Blond	1.17 (0.94–1.46)	0.15	1.07 (0.92–1.24)	0.36
Medium	1.06 (0.93–1.22)	0.35	1.11 (0.99–1.24)	0.09
Dark	1.87 (1.29–2.72)	<0.001	0.78 (0.64–0.95)	0.01

Multivariable analysis was adjusted by fundus pigmentation and factors significant in univariable analysis.

a Adjusted by male sex, use of UC3 probe, and gestational age.

b Adjusted by use of UC3 probe, gestational age, and birth weight.

## Discussion

To our knowledge, this is the first study to evaluate the association between fundus pigmentation and visibility of retinal versus choroidal layers on bedside OCT in preterm infants. This study found that fundus pigmentation was not associated with the visibility of all retinal layers. However, interestingly, older age at imaging was associated with increased retinal layer visibility, specifically among infants with dark fundus pigmentation. This was an unexpected finding that could potentially suggest decreased retinal layer visibility among younger infants with dark fundus pigmentation. However, retinal layer visibility at the youngest ages (i.e., <32 weeks’ PMA) among infants with dark pigmentation was comparable to or better than that among those with blonde or medium pigmentation. We therefore hypothesize that the observed association results from the very high retinal layer visibility among infants with dark fundus pigmentation at older ages, rather than poor visibility at younger ages.

This study also found that medium and dark fundus pigmentation was associated with decreased CSJ visibility on OCT. We hypothesize that the more prominent choroidal melanin in infants with darker fundus pigmentation limits the penetration of OCT signal to, and therefore visibility of, deeper structures (i.e., the CSJ) but does not impact penetration to more inner structures (i.e., other retinal layers). In our study, among infants with dark pigmentation, CSJ visibility decreased with increasing age at OCT imaging. This decreased visibility over time likely correlates to the development of choroidal pigmentation^22^^,^^23^ after preterm birth and provides further support for the direct impact of choroidal pigmentation on the visibility of the CSJ on bedside OCT in preterm infants. An alternative explanation for decreasing CSJ visibility over time could be choroid thickness, which increases significantly between 30 and 38 weeks’ PMA in preterm infants.^34^ However, increasing choroid thickness alone is unlikely to account for the observed decrease in CSJ visibility over time in infants with dark pigmentation, because CSJ visibility did not change with increasing age in infants with blond or medium pigmentation. An important strength of this study is the longitudinal evaluation of the visibility of retinal and choroidal layers, rather than a cross-sectional evaluation at a single time point.

Previous work by Moreno and colleagues^27^ investigated the association between self-reported maternal race and CSJ visibility on bedside spectral-domain OCT (research model of Envisu 2300; Bioptigen, Morrisville, NC) at 840-nm central wavelength in 86 preterm infants, term infants, and adults. Their study found that African American race was associated with decreased CSJ visibility in all age groups (except the youngest preterm infant ages, 30–36 weeks’ PMA), and concluded that increased choroidal pigmentation in African Americans resulted in poorer OCT signal penetration and CSJ visibility. At 30 to 36 weeks’ PMA, CSJ visibility was >90% for each race, suggesting that choroidal pigmentation was incomplete at preterm birth and that development of pigmentation over time in African Americans resulted in decreased CSJ visibility at older ages.^27^ Note that the relevant biological variable of fundus pigmentation was confounded by the use of the sociological identification of race. We hypothesize that darker fundus pigmentation mediates the association between African American maternal race and decreased CSJ visibility on OCT imaging, which would explain the consistency between our findings and those of Moreno and colleagues.^27^ Further improvements in future OCT imaging may improve CSJ visibility among infants with dark fundus. Notably, in our cohort, maternal race was a sensitive, but not specific, marker for dark fundus pigmentation; that is, although all infants with dark pigmentation were non-White, not all non-White infants had dark pigmentation (some non-White infants had blond or medium pigmentation). The direct assessment of the impact of fundus pigmentation on CSJ visibility, rather than the use of maternal race as an imperfect proxy measure, is an advantage of our study over previous work.

Our study also identified several factors, beyond fundus pigmentation, that independently impacted retinal and choroidal layer visibility on bedside OCT. First, greater birth weight was independently associated with increased odds of all retinal layers being visible. We hypothesize that this is due to the greater restrictive requirements when approaching sicker infants (e.g., greater nurse concern for research imaging or presence of face mask or other supportive devices). Second, use of the UC3 probe was associated with increased odds of all retinal layers and the CSJ being visible. This is likely due to several improvements in the design of the system from UC2 to UC3, resulting in overall better OCT image quality. Notably, although mean pupil size at imaging was smaller in infants with dark fundus pigmentation than in infants with blond or medium pigmentation, there was no association between pupil size at OCT imaging and visibility of all retinal layers or the CSJ. This finding suggests that it is truly darker fundus pigmentation, rather than poorer pupil dilation in infants with a darker fundus and iris, that accounts for the lower CSJ visibility in infants with dark fundus pigmentation. This finding also further supports prior claims that bedside OCT can reliably capture retinal microanatomy in preterm infants without the need for pharmacological dilation.^35^

This study has several limitations. First, this study lacked inclusion of many infants outside of two racial groups (Black or African American and White). Second, there were relatively few eyes with dark fundus pigmentation. Third, because the quality of en face imaging changed with pixel density midway through the study, we analyzed cross-sectional OCT scans and not the quality of en face posterior pole retinal vessel maps. Thus, although the demonstrated visibility of retinal layers, particularly the RPE, should be sufficient for the generation of high-quality retinal vessel maps,^20^ we could not directly assess the impact of fundus pigmentation on en face retinal vessel map quality.

In conclusion, this study found that fundus pigmentation was not associated with visibility of all retinal layers, whereas darker fundus pigmentation was associated with decreased CSJ visibility on bedside OCT. Among infants with dark fundus pigmentation, CSJ visibility significantly decreased on OCT with increasing age, corresponding to the development of choroidal pigmentation after preterm birth. All retinal layers were visible in the vast majority of OCT scans overall, and at all ages and pigmentation levels. These findings suggest that investigational bedside OCT can capture retinal layer microanatomy in diverse populations of preterm infants, including those with dark fundus pigmentation. Future studies of image quality of OCT en face retinal vessel maps and contact fundus photography in infants with a range of fundus pigmentation are needed.
